# Supplementary material for: Drosophila as Model System to Study Ras-Mediated Oncogenesis: The Case of the Tensin Family of Proteins
Source: Genes (Basel). 2023 Jul 23;14(7):1502. doi: 10.3390/genes14071502 (PMC10379045; doi:10.3390/genes14071502)
Supplement: Supplementary file 1 [file genes-14-01502-s001.zip › genes-2519781-supplementary.pdf]

Supplementary Figure Legends.

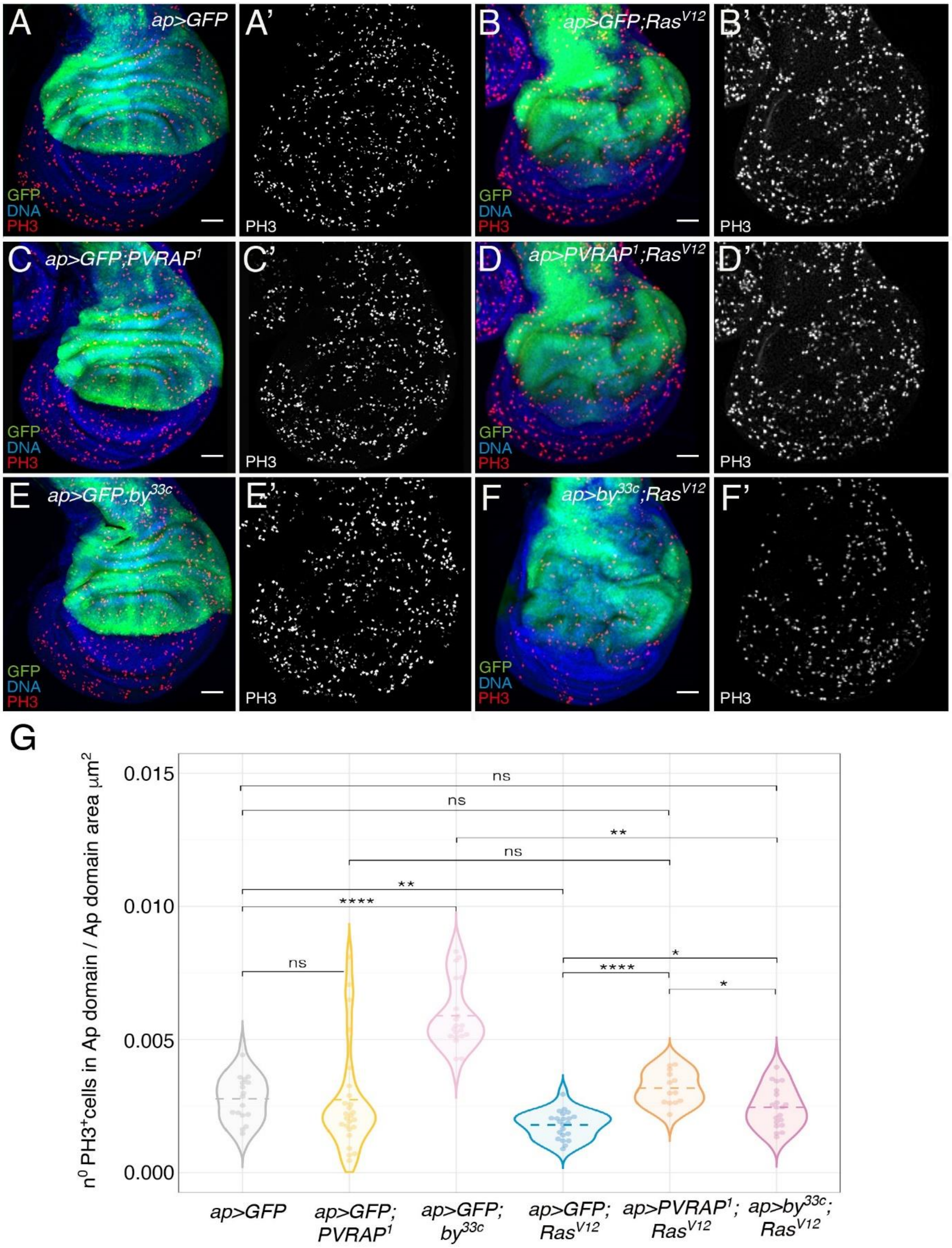

Supplementary Figure S1: Elimination of *by* or *PVRAP* increases cell proliferation in Ras^{V12} expressing wing discs. (A-F) Maximal projection of confocal views of 3rd instar larvae

wing discs, expressing GFP (green) and the indicated UAS transgenes under the control of *apGal4*, in control and mutant backgrounds, stained with anti-GFP (green), PH3 (red) and Hoechst (DNA, blue). (G) Violin plots of the number of PH3+ cells per GFP area (G). The statistical significance of differences was assessed with a welch-test, ****P value<0.0001. Scale bars 50 µm.

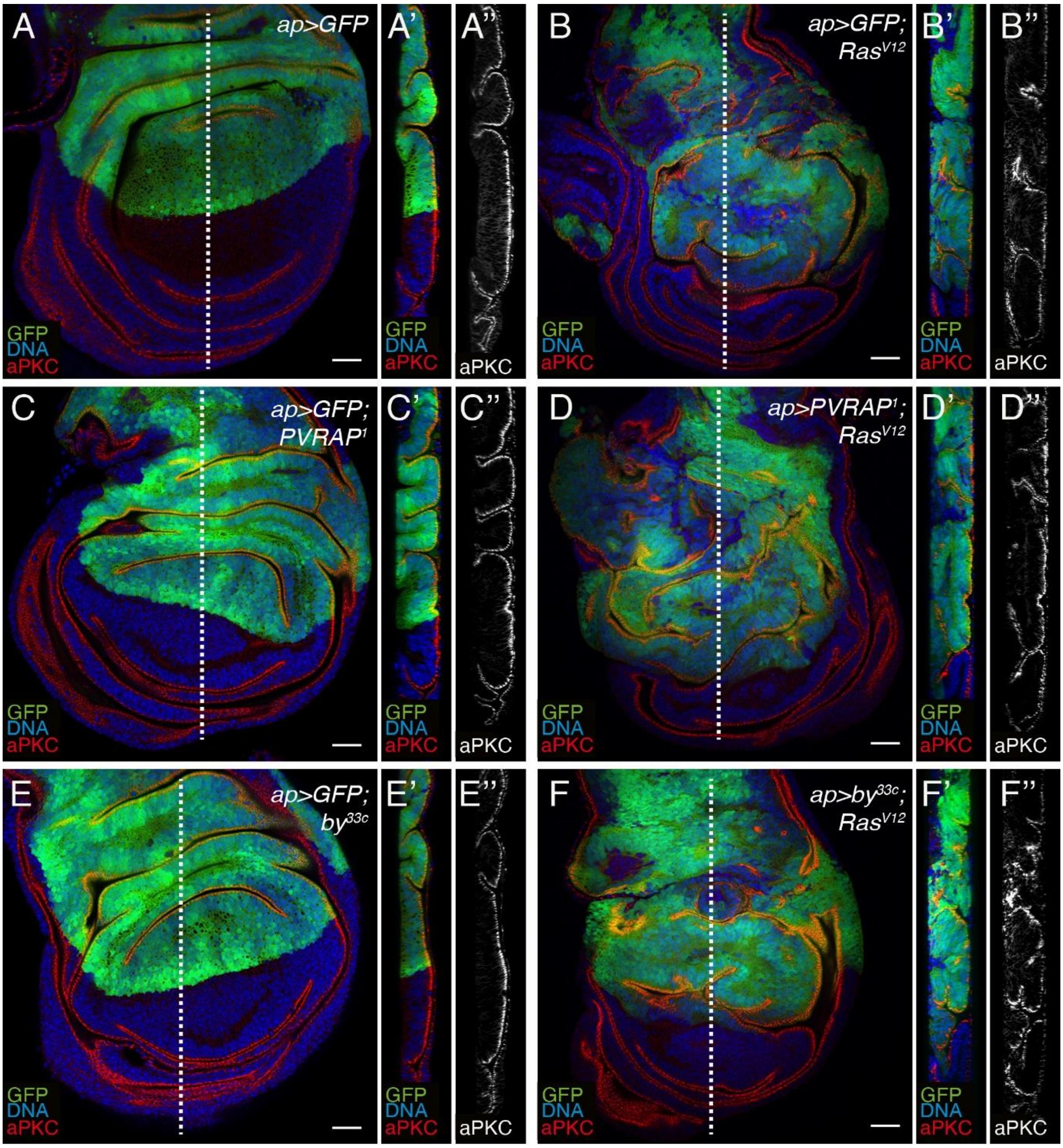

Supplementary Figure S2: Cell polarity is not affected by the removal of *by* or *PVRAP* either in normal or Ras^{V12} conditions. (A-F) Confocal images of 3rd instar wing discs of the

indicated genotypes stained with anti-GFP (green), anti-aPKC (red) and Hoechst (DNA, blue). (A'-F' and A''-F'') Confocal *xz* sections along the white dotted lines of wing discs shown in A-F. The apical side of wing discs is at the right. Scale bars, 30 μm (A-F).

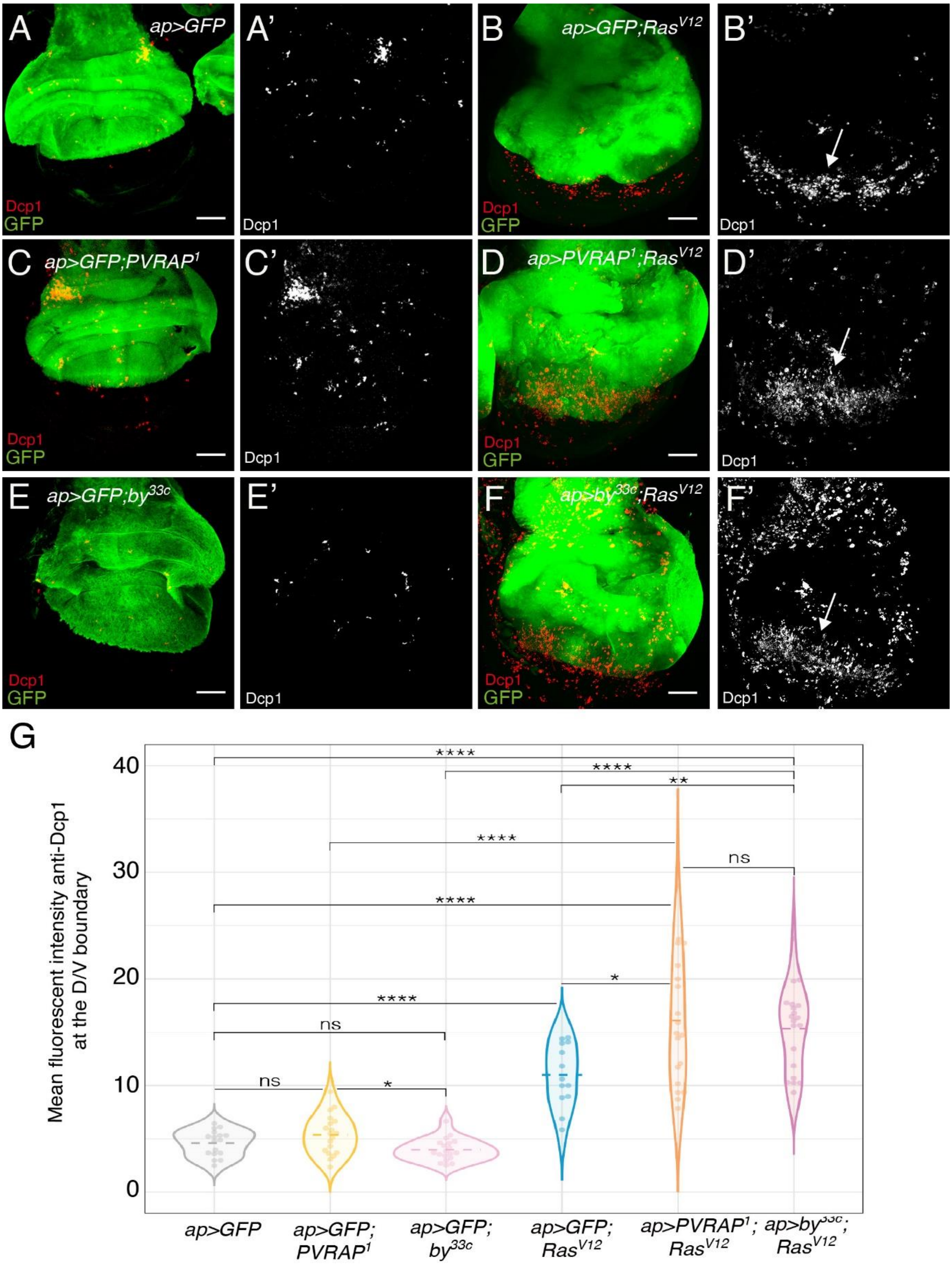

Supplementary Figure S3: Apoptosis of nearby wild type tissue due to Ras^{V12} overexpression is not affected by elimination of wither *by* or *PVRAP*. (A-F) Maximal projection of confocal views of wing imaginal discs from third-instar larvae expressing GFP (green) and the indicated UAS transgenes under the control of *apGal4*, stained with anti-GFP (green), anti-Dcp-1 (red in A-F, white in A'-F') and Hoechst (DNA, blue). (G) Violin plots of mean fluorescent Dcp-1 intensities at the D/V boundary of wing discs with the designated genotypes. The statistical significance of differences was assessed with a welch-test, **** and *** P values <0.0001 and <0.001, respectively. Scale bars 50 µm.

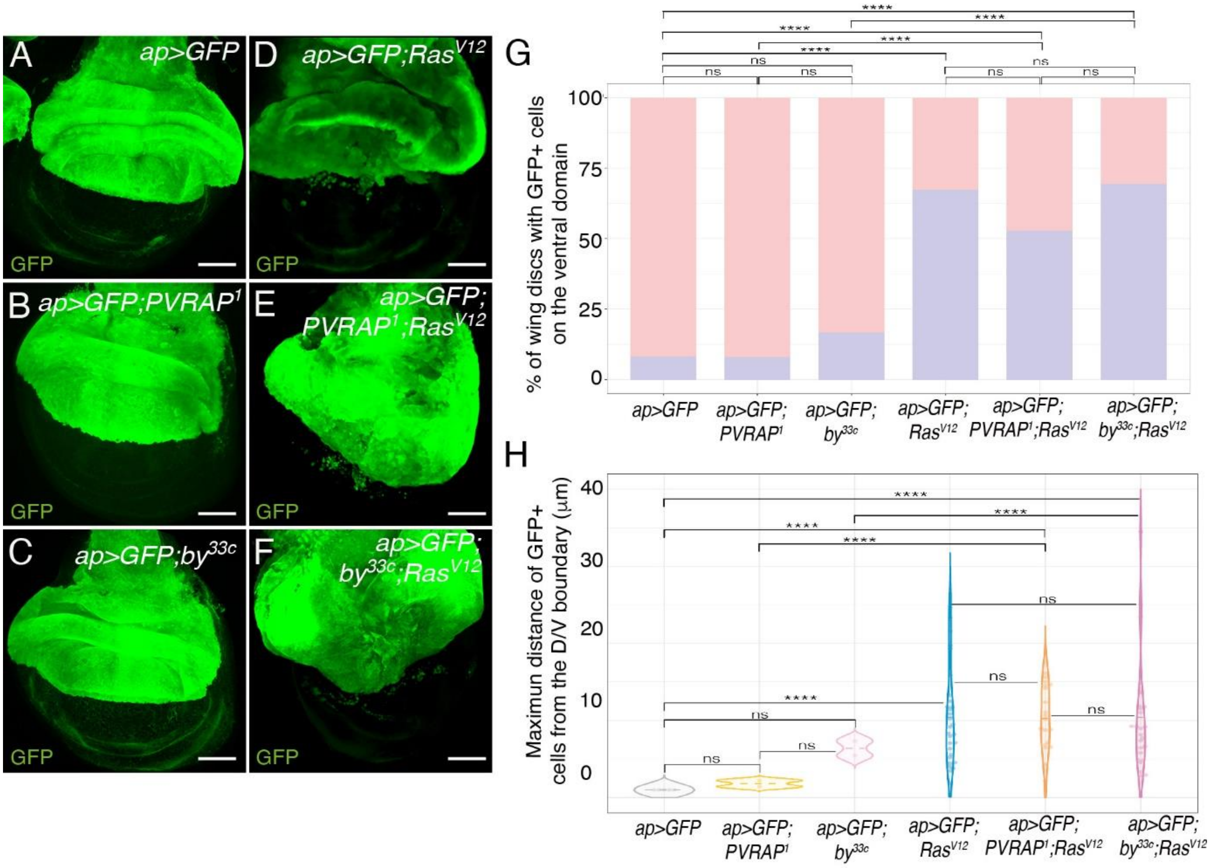

Supplementary Figure S4: Elimination of either *by* or *PVRAP* does not affect the migratory capacity of Ras^{V12} wing disc expressing cells. (A-F) Maximal projection of confocal views of 3rd instar wing discs expressing GFP (green) and the indicated UAS transgenes under the control of *apGal4*, stained with anti-GFP (green). (G) Bar plot representing the presence or absence of GFP^{+} cells in the ventral domain. (H) Violin plots showing the distance at which GFP^{+} cells are found with respect to the D/V boundary. The statistical significance of differences was assessed with a xi-square (G) and welch-test (H), **** P values <0.0001. Scale bars 50 µm.
